# Absorbable Versus Silk Sutures for Surgical Treatment of Trachomatous Trichiasis in Ethiopia: A Randomised Controlled Trial

**DOI:** 10.1371/journal.pmed.1001137

**Published:** 2011-12-13

**Authors:** Saul N. Rajak, Esmael Habtamu, Helen A. Weiss, Amir Bedri Kello, Teshome Gebre, Asrat Genet, Robin L. Bailey, David C. W. Mabey, Peng T. Khaw, Clare E. Gilbert, Paul M. Emerson, Matthew J. Burton

**Affiliations:** 1 The London School of Hygiene and Tropical Medicine, London, United Kingdom; 2 The Carter Center, Addis Ababa, Ethiopia; 3 Light For The World, Addis Ababa, Ethiopia; 4 The Amhara Regional Health Bureau, Bahir Dar, Ethiopia; 5 NIHR Biomedical Research Centre for Ophthalmology, Moorfields Eye Hospital and UCL Institute of Ophthalmology and UCL Partners AHSC, London, United Kingdom; 6 The Carter Center, Atlanta, United States of America; Kilimanjaro Centre for Community Ophthalmology, Tanzania

## Abstract

**Background:**

Trachoma causes blindness through an anatomical abnormality called trichiasis (lashes touching the eye). Trichiasis can recur after corrective surgery. We tested the hypothesis that using absorbable sutures instead of silk sutures might reduce the risk of recurrent disease among patients with major trichiasis in a randomised trial.

**Methods and Findings:**

1,300 individuals with major trichiasis from rural villages in the Amhara Region of Ethiopia were recruited and assigned (1∶1) by computer-generated randomisation sequence to receive trichiasis surgery using either an absorbable suture (polyglactin-910) or silk sutures (removed at 7–10 days) in an otherwise identical surgical technique. Participants were examined every 6 months for 2 years by clinicians masked to allocation. The primary outcome measure was recurrent trichiasis (≥one lash touching the eye) at 1 year. There was no difference in prevalence of recurrent trichiasis at 1 year (114 [18.2%] in the absorbable suture group versus 120 [19.7%] in the silk suture group; odds ratio = 0.90, 95% CI 0.68–1.20). The two groups also did not differ in terms of corneal opacification, visual acuity, conjunctival inflammation, and surgical complications.

**Conclusions:**

There was no evidence that use of absorbable polyglactin-910 sutures was associated with a lower prevalence of trichiasis recurrence at 1 year postsurgery than silk sutures. However, from a programmatic perspective, polyglactin-910 offers the major advantage that patients do not have to be seen soon after surgery for suture removal. The postoperative review after surgery using absorbable polyglactin-910 sutures can be delayed for 3–6 months, which might allow us to better determine whether a patient needs additional surgery.

**Trial registration:**

ClinicalTrials.gov NCT00522860

*Please see later in the article for the Editors' Summary*

## Abstract 2

In this randomized trial, Saul Rajak et al. compare silk sutures (removed at 7–10 days) or absorbable sutures (left in place) during surgery for the management of trachomatous trichiasis.

## Editors' Summary

**Background:**

Globally, around 40 million people—mostly people living in rural areas in developing countries where there are water shortages, poor personal hygiene, and crowded living conditions—have active trachoma, an infectious eye disease that is caused by *Chlamydia trachomatis*. This bacterium is spread through contact with infected eye secretions or with contaminated towels or clothes, and by flies. Recurrent infections with *C. trachomatis* during early childhood cause inflammation of the tissue lining the eye lid (chronic conjunctival inflammation), which can lead to conjunctival scarring. If this scarring is severe, the eyelids turn inwards (entropion) and the lashes rub across the eye's surface (the cornea). This condition—trachomatous trichiasis—is extremely painful and, if not treated with surgery, can lead to irreversible corneal opacities and visual impairment by middle age. It is estimated that 8 million people have trichiasis and that an additional 8 million people are blind or visually impaired as a result of the condition.

**Why Was This Study Done?:**

Surgery for trichiasis, antibiotics for infection, and facial cleanliness and environmental improvements to reduce transmission together constitute the SAFE strategy for the control of blinding trachoma. Unfortunately, trichiasis recurs in nearly two-thirds of patients within 3 years of surgery, often within the first year. How the surgery is performed, its quality, and the severity of entropion and conjunctival scarring at the time of surgery all contribute to trichiasis recurrence. In this randomized trial (a study in which randomly chosen groups of patients receive different treatments for a disease and are followed to compare the outcomes of these interventions), the researchers investigate whether using absorbable sutures instead of silk sutures reduces the risk of recurrent disease among patients with major trichiasis (more than five lashes touching the cornea). Sutures are used to sew up surgical incisions. Silk sutures, which are used routinely during trichiasis surgery, have to be removed 7–10 days after surgery when the incision may not have stably healed. Absorbable sutures might provide more stable fixation of the eye tissue while healing is taking place and might, therefore, reduce the recurrence of trichiasis after surgery.

**What Did the Researchers Do and Find?:**

The researchers randomly assigned 1,300 people living in Ethiopia (the country with the highest rates of trachoma and trichiasis) to receive trichiasis surgery using silk sutures (removed at 7–10 days) or absorbable sutures (left in place); the other details of the surgery were identical for all the patients. The trial's primary outcome was recurrent trichiasis (one or more lash touching the eye) at 1 year. Secondary outcomes included the rate of recurrence, visual acuity, corneal opacity, and conjunctival inflammation at 2 years, and surgical complications. At 1 year, 18.2% of the patients in the absorbable suture group and 19.7% in the silk suture group had developed recurrent trichiasis. That is, the prevalence of recurrence in the two groups was similar. There was also no difference in the rate of trichiasis recurrence between the groups 2 years after surgery. Moreover, the two groups did not differ in terms of corneal opacity and visual acuity, conjunctival inflammation, or surgical complications.

**What Do These Findings Mean?:**

These findings provide no evidence to suggest that the use of absorbable sutures during trichiasis surgery is clinically better than the use of silk sutures. However, the researchers note that the use of absorbable sutures would eliminate the need for patients to return to the clinic soon after their operation to have their sutures removed. In remote rural settings, it can be difficult for patients, who are often elderly and poor, to attend clinics. Thus, it might be better for trachoma services to concentrate on encouraging patients to return 3–6 months after surgery when the need for additional surgery can be determined rather than trying to encourage them to come back after 7–10 days for suture removal. The use of absorbable sutures would also avoid any complications arising from patients failing to come back to have their stitches removed. The researchers suggest, therefore, that trachoma control programs should now consider the potential logistical advantages of using absorbable sutures rather than silk sutures during trichiasis surgery despite the lack of any apparent clinical difference between the two suture types.

**Additional Information:**

Please access these Web sites via the online version of this summary at http://dx.doi.org/10.1371/journal.pmed.1001137.

- An accompanying PLoS Medicine Research Article by Saul Rajak et al. describes another randomized trial undertaken by these researchers that compares surgery and epilation (eyelash removal) for the treatment of trichiasis in Ethiopia
- The World Health Organization has information on trachoma (in several languages), including details of the Alliance for Global Elimination of Trachoma by the year 2020 (GET 2020) and a personal story about blinding trachoma
- The UK National Health Service Choices website also provide information on trachoma
- Orbis, an international nonprofit organization devoted to blindness prevention and treatment in developing countries, provides information about trachoma
- The International Trachoma Initiative provides detailed information about trachoma and a personal story about trichiasis surgery in Ethiopia
- The Global Atlas of Trachoma is an open-access resource on the geographical distribution of trachoma
- Light for the World is a nonprofit organization dedicated to ensuring the rights of persons with disabilities in developing countries, including people in Ethiopia with trachoma

## Introduction

Trachoma is the leading infectious cause of blindness worldwide [1]. Recurrent episodes of ocular *Chlamydia trachomatis* infection in early childhood provoke chronic conjunctival inflammation (active trachoma). This inflammation leads to scarring of conjunctival tissue, which causes the eyelids to roll in (entropion) and the lashes to scratch the surface of the eye (trachomatous trichiasis [TT]). Ultimately, blinding corneal opacification (CO) develops from the persistent abrasion of lashes and secondary bacterial infection.

Blinding trachoma is reported to be prevalent in more than 50 countries [1]. Over 40 million individuals (mostly children) are estimated to have active trachoma at any one time, 8 million people have trichiasis and a further 8 million are estimated to be blind or visually impaired from the disease [1],[2]. Ethiopia has the highest rates of active trachoma and trichiasis in the world, with an estimated 1.3 million unoperated cases of TT [1]. Endemic countries are striving to control this disease through the implementation of the SAFE strategy: surgery for trichiasis, antibiotics for infection, facial cleanliness and environmental improvements to reduce transmission [3].

To prevent blindness from trichiasis, surgery is performed to correct the entropion, lifting the eyelashes off the cornea. Ophthalmic services are generally limited in trachoma endemic settings; therefore, specially trained nurses usually perform the surgery in the community. Several different procedures have been tried over the last century [4], but trichiasis frequently recurs either because of some intrinsic limitation or quality of the surgery, or because of progressive scarring. Studies of trichiasis recurrence rates following surgery conducted under “operational” conditions have consistently shown disappointing outcomes, with usually at least 20% recurrence by 1 y and up to 62% at 3 y [5]–[11]. Moreover, these poor surgical outcomes undermine other efforts to prevent blindness from trachoma. Context-appropriate interventions to improve results are urgently needed.

Several factors contribute to recurrent trichiasis, which can be divided into early surgery-related and later disease-related factors. Data from prospective studies suggest that the majority of recurrent trichiasis develops within 6 mo of surgery, indicating the importance of how the surgery is performed [5],[8],[9],[12]–[14]. Randomised controlled trials (RCTs) of alternative procedures indicate that bilamellar tarsal rotation (BLTR) and posterior tarsal rotation (PLTR) procedures are associated with the lowest recurrence rates, leading to the World Health Organization's (WHO) recommendation of their use [3],[4],[12],[15],[16]. However, recurrence rates for both procedures are quite high [5]–[8],[17]–[19]. The quality of surgery is important, indicated by significant variation in the results of different surgeons [8].

Suture type, positioning, and tension are likely to be important aspects of surgical technique that contribute to TT recurrence. Silk sutures are used as standard in trichiasis surgery and need to be removed 7–10 d postoperatively [16]. At this stage the incision may not have reached a state of stable wound healing, because of the scarred nature of the diseased tissue. The use of absorbable suture materials, such as polyglactin-910, which is commonly used in ophthalmic and other surgery, may provide more prolonged and stable fixation of the tissue in the desired position while healing is taking place [20].

A retrospective review of surgical outcomes in Egypt found that recurrence rates were significantly lower in individuals who had received long-lasting absorbable sutures (0.8%) compared to those with silk sutures (43.5%) [11]. In this current study, we test the hypothesis that using absorbable polyglactin-910 sutures can reduce the postoperative trichiasis recurrence rate compared to the current standard silk sutures, in a randomised trial in Amhara Region, Ethiopia.

## Methods

### Ethics Statement

The National Health Research Ethics Review Committee of the Ethiopian Ministry of Science and Technology, the London School of Hygiene and Tropical Medicine Ethics Committee, and Emory University Institutional Review Board approved the trial. Potential participants were provided with both written and oral information in Amharic about the trial. For those agreeing to participate, written informed consent in Amharic was required prior to enrolment. If the participant was unable to read and write, the information sheet and consent form were read to them and their consent recoded by witnessed thumbprint. An independent Data Safety Monitoring Committee reviewed the trial for patient safety and there were no deviations from the original protocol. No interim analyses for efficacy or futility were planned or conducted. The Trial Protocol is described in Text S1 and the CONSORT statement in .

### Participants

Eligible participants were individuals aged 18 y or over with previously unoperated major trichiasis (>five trichiatic eyelashes, some of which may have been epilated and were regrowing) who presented during a TT surgical treatment campaign in rural villages in the West Gojjam zone of the Amhara Region of Ethiopia from March to June 2008. Exclusion criteria were previous eyelid surgery, medically unfit, or pregnancy (self-reported or clearly evident). The surgical campaigns were advertised in local markets, churches, and schools. Additionally, health extension workers from the subdistricts (*kebele*) in the study area were trained to recognize trichiasis and visited each village in their *kebele* to identify patients.

### Preoperative Clinical Assessment

The clinical assessments and surgery were performed in government health centres, clinics, and other suitable buildings. A field worker administered a questionnaire in Amharic. Height and weight were measured. Unaided and pinhole LogMAR visual acuities were measured at 4 m, using an ETDRS equivalent Tumbling-E LogMAR chart (Hong Kong Low Vision Centre). The testing distance was reduced to 2 or 1 m if necessary. For those unable to read the LogMAR chart at 1 m their visual acuity was tested by counting fingers at 1 m or hand movements at 0.5 m. For visual acuities of counting fingers or less, LogMAR values were attributed: counting fingers, 2.0; hand movements, 2.5; perception of light, 3.0; no perception of light, 3.5. No patients had spectacles for distance correction. Ophthalmic examinations were conducted in a darkened room using 2.5× magnification loupes and a bright torch. A single ophthalmologist (SNR) performed all baseline examinations. The number of lashes touching the eye was counted (“lash burden”) and also subdivided by the part of the eye contacted when looking straight ahead: cornea, lateral conjunctiva, or medial conjunctiva. Clinical evidence of epilation was identified by the presence of broken or newly growing lashes, or areas of absent lashes. Upper lid entropion was graded by assessing the degree of inward rotation of the eyelid margin (Table S1). The degree of corneal scarring was classified on the basis of a modified WHO FPC grading system in which the degree of central corneal scarring (CC2) is subdivided to provide more definition (Table S1) [21]. Tarsal conjunctival papillary inflammation, follicles, and scarring were classified using the WHO FPC grading system [21]. Standardised high-resolution digital photographs were taken of each of these features.

### Interventions

Following the preoperative clinical assessment, participants were randomised to one of two surgical intervention groups, which differed only in the type of sutures that were used. The posterior lamella tarsal rotation procedure was used in all cases [5]. Surgery was performed under local anaesthesia administered by subcutaneous infiltration of the upper eyelid: 2–3 ml of lidocaine 1%, with adrenaline. The lid was then everted and the posterior lamella (tarsal conjunctiva and tarsal plate) incised parallel to and 3 to 4 mm above the lid margin. The posterior lamella was separated from the anterior lamella (obicularis oculi and skin) by blunt dissection in the tissue plane. Three sets of everting sutures were placed running down and forward from the upper cut edge of the tarsal plate to emerge through the skin just above the eyelashes. As the sutures are tightened they externally rotate the lower border of the eyelid. The sutures were either (1) silk, 4/0 (Mersilk, Ethicon) or (2) polyglactin-910, 5/0 (Vicryl undyed, Ethicon). All sutures had three-eighth circle, 16-mm cutting needles at each end (“double ended”). Postoperatively, the operated eye was padded for a day and then tetracycline eye ointment was self-administered twice a day for 2 wk. If the participant had bilateral trichiasis they were offered surgery to both eyes on the day of enrolment, using the same randomly allocated suture type for each eye.

Five nurses, who had previously been trained in and were regularly performing TT surgery, performed the surgery in this trial. They were selected as the best surgeons from a larger group of nine during a 2-d standardisation workshop. The training was conducted by an experienced Ethiopian ophthalmologist (ABK), who had contributed to the development of the WHO TT surgeon certification manual [22]. The posterior lamellar tarsal rotation techniques of the five nurses were carefully observed and standardised to ensure that all performed the operation in the same way.

### Follow-up Clinical Assessments

All participants were seen 7–10 d postoperatively, when silk sutures were removed and any complications noted and treated as needed. Follow-up assessments were conducted at 3, 6, 12, 18, and 24 mo postoperatively. On each occasion an ophthalmic examination was performed and digital photographs taken. Photographs were not taken at 3 mo. Visual acuity was measured at 12 and 24 mo. The 12-mo and 24-mo follow-up examinations were conducted by the same single ophthalmologist (SNR) who performed the preoperative examinations; the 3-, 6-, and 18-mo examinations were conducted by a single ophthalmic nurse (EH). Neither of these examiners was involved in performing surgery in this trial. The examiners were standardised to each other and showed strong agreement between these two observers for the presence of trichiasis in a preliminary assessment of 200 eyes (kappa = 0.86) Several different ophthalmic nurses who did not take part in subsequent follow-ups conducted the 7-d follow-up. Any individual who developed significant recurrent trichiasis during the follow-up period was offered repeat surgery performed by a senior surgeon. These reoperated individuals continued to be followed up according to the trial protocol. Individuals in whom other ophthalmic pathology (e.g., cataract) was detected were referred to the regional ophthalmic services.

### Outcome Measures

The primary outcome measure was the proportion of those individuals seen at the 12-mo follow-up who were found to have either (1) recurrent trichiasis, defined as one or more lashes touching the eye or clinical evidence of epilation, or (2) a history of repeat TT surgery during the first year. Individuals not seen at 12 mo were excluded from this primary analysis. A priori defined secondary outcome measures at 12 and 24 mo were: CO, visual acuity, entropion, and conjunctival inflammation. Change in CO was determined both from direct comparison of the 1- and 2-y photographs with the baseline photographs and from the field grading scores. This enabled us to detect progression or regression using an alternative method of assessment of corneal opacities.

### Randomisation, Allocation Concealment, and Masking

Participants were randomly allocated to the silk or polyglactin-910 suture arms using a 1∶1 allocation ratio for each surgeon, using a computer-generated randomisation sequence with random block sizes. Randomisation was stratified by surgeon because of possible intersurgeon variability (each surgeon had a separate sequence). The statistician held the master randomisation lists. The random allocation sequences for each surgeon were concealed in sequentially numbered, sealed, opaque envelops, which were placed in separate containers for each surgeon; a person independent of all other aspects of the trial prepared these envelopes. On most days during the surgery campaign two or three surgeons operated simultaneously. Following the baseline examination, participants were allocated to the next available surgeon. A trained field worker was responsible for implementing the intervention assignment in a dedicated area, separated from those performing the preoperative examinations. The field worker and surgeon jointly confirmed the suture allocation and recorded this in the surgical logbook. The same field worker dispensed the appropriate sutures to the surgeon. Participants and surgeons were aware of the suture allocation. The two individuals (SNR, EH) who were responsible for all the clinical observations in this trial were masked to the allocation. For the purposes of the trial analysis only one eye per person was considered. If the participant had bilateral trichiasis operated on, the study eye (for analytical purposes) was chosen at random using a computer generated randomisation list.

### Statistical Methods

Using a conservative 1-y risk of TT recurrence of 10% in the silk arm we considered a 50% reduction in recurrence to 5% in the polyglactin-910 arm to be programmatically significant [8],[13]. A sample of 1,240 participants (620 in each arm) would provide 90% power and 95% confidence to detect a difference with a relative risk of 0.5; this was increased to 1,300 to allow for 5% loss to follow-up.

Data were double entered into an Access (Microsoft) database and transferred to Stata 11 (StataCorp) for analysis. The analysis was on an intention-to-treat basis. For participants who had bilateral surgery only the randomly designated study eye was included in the analysis. We made every effort to obtain all outcome data for every participant. We excluded patients from analyses if they had missing data relevant to a particular analysis, as indicated in the results. Missing data were not imputed.

The primary outcome (recurrent trichiasis at 12 mo) and secondary outcomes were compared between the two groups using logistic regression analyses to estimate the odds ratio (OR) and 95% CI. Time to recurrence was analysed with Kaplan-Meier survival curves, and Cox regression models were used to estimate hazard ratios (HRs) and associated 95% CI. Individuals who had reached endpoint at a previous time point or were permanently lost to follow-up from that time onwards were censored. A multivariable logistic regression model was developed to estimate OR and 95% CI for associations of any recurrent trichiasis during the 2 y of follow-up with potential explanatory factors. Variables that were associated with recurrent trichiasis on univariate analysis (*p*<0.2) were retained in the multivariable model. The surgeon with the lowest recurrence rate at 2 y was used as the reference for the others in the models.

## Results

### Participant Recruitment and Flow

**Figure 1:**
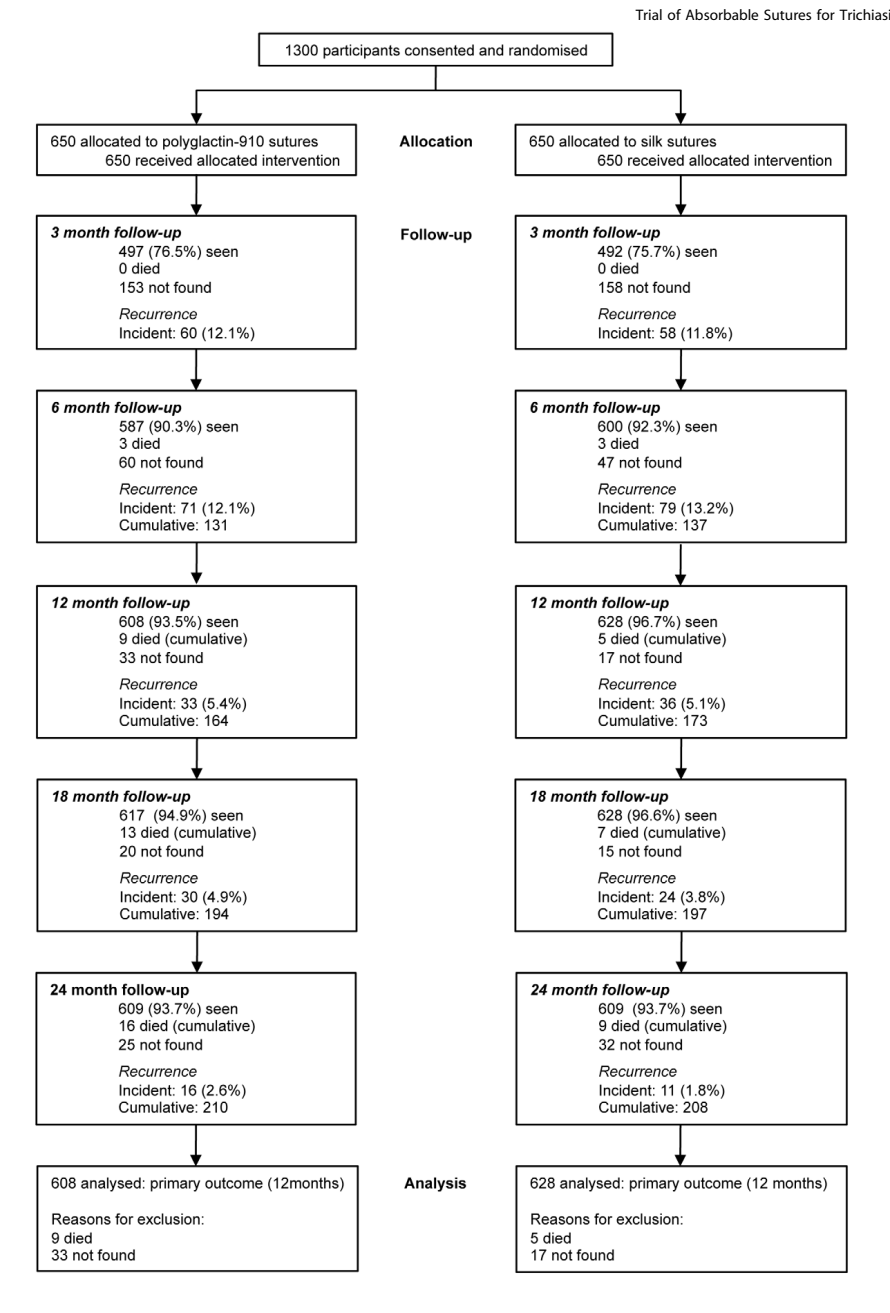
Trial profile.

Recruitment ran from March–June 2008. Several thousand people presented with eye complaints during the course of the surgical outreach campaign and were assessed for eligibility for this trial. The majority had other ophthalmic conditions such as cataract. It was not possible to record the total number of individuals examined who did not have trichiasis. A total of 1,300 consecutive individuals who met the inclusion criteria were recruited; none were excluded and all consented and were enrolled in the trial. All 1,300 were randomised and received their allocated surgical treatment (650 participants in each trial arm) (Figure 1).

Primary outcome data are available for 608/650 (93.5%) participants in the silk group and 628/650 (96.7%) of participants in the polyglactin-910 group (Figure 1). Overall, 69.3%% of participants were seen at all five scheduled visits over 24 mo (447/650 [68.8%] in the silk group and 454/650 [69.9%] in the polyglactin-910 group). At all follow-up time points more than 90% of participants were examined, except for the 3-mo follow-up, which was limited by the wet season. There was little difference by randomisation group in the number of times individuals were seen during the follow-up (*p* = 0.60) or the number of participants seen at each follow-up (Figure 1). All 1,300 participants were reviewed at 7 to 10 d. No participants in the silk arm were recorded as not having their sutures removed at 7–10 d. However, it was possible that incomplete removal could have caused suture fragments to be left in situ. In these few participants the individual conducting the participant's next follow-up examination was unmasked. 15 participants were not seen again between 3 and 24 mo after surgery and were excluded from all analyses: 11 from the silk arm and four from the polyglactin-910 arm.

### Baseline Demographic and Clinical Characteristics

Baseline sociodemographic characteristics were balanced by randomisation group (Table 1). All participants were Ethiopians of Amharan ethnicity. Their mean age was 49.8 y and the majority (78.1%) were female. Literacy rates and nutritional status (body mass index) were comparable. Clinical features were also balanced by randomisation group (Table 1). The baseline field and photographic CO scores showed good correlation (linear weighted kappa score 0.77; quadratic weighted kappa score 0.88; similar agreement at 12 and 24 mo).

**Table 1:** Baseline characteristics and number of follow-up assessments of participants, by study arm.

Characteristic	Silk, *n* = 650		Polyglactin-910, *n* = 650	
	*n*	Percent or 95% CI	*n*	Percent or 95% CI
***Gender (female)***	508	(78.2%)	507	(78.0%)
***Age***				
18–29	48	(7.4%)	60	(9.2%)
30–39	123	(18.9%)	127	(19.5%)
40–49	186	(28.6%)	159	(24.5%)
50–59	167	(25.7%)	160	(24.6%)
60–69	93	(14.3%)	112	(17.2%)
70+	33	(5.1%)	32	(4.9%)
Mean (SD, 95% CI)	49.7	(12.9, 48.7–50.7)	49.9	(13.6, 48.9–51.0)
***Illiterate***	617	(94.9%)	625	(96.2%)
***BMI***, mean (SD, 95% CI)	20.1	(2.5, 19.9–20.3)	19.8	(2.3, 19.6–20.0)
***Study eye (right)*** a	321	(49.4%)	352	(54.2%)
***Best corrected LogMAR VA in study eye***				
−0.2 to 0.3	193	(30.0%)	203	(31.6%)
0.3–0.7	248	(38.5%)	250	(38.9%)
0.7–1.1	104	(16.2%)	95	(14.8%)
1.1–2.0	29	(4.5%)	25	(3.9%)
CF/HM/PL	62	(9.6%)	59	(9.2%)
NPL	8	(1.2%)	10	(1.6%)
Not measurable^b^	6		8	
***Entropion grade***				
0	51	(7.9%)	69	(10.6%)
1	159	(24.5%)	153	(23.5%)
2	242	(37.2%)	248	(38.2%)
3	119	(18.3%)	98	(15.1%)
4	79	(12.2%)	82	(12.6%)
***Trichiasis (number of lashes touching eye)***				
None (epilating)	106	(16.3%)	98	(15.1%)
1–5 (epilating)	253	(38.9%)	295	(45.4%)
6–9	152	(23.4%)	131	(20.2%)
10–19	89	(13.7%)	72	(11.1%)
20+	50	(7.7%)	54	(8.3%)
***Lower lid TT (present)***	88	(13.5%)	85	(13.1%)
***Corneal opacity***				
CC0, none	187	(28.8%)	194	(29.9%)
CC1, peripheral	181	(27.9%)	201	(30.9%)
CC2a, off centre faint	147	(22.6%)	119	(18.3%)
CC2b, off centre dense	26	(4.0%)	24	(3.7%)
CC2c, central faint	63	(9.7%)	68	(10.5%)
CC2d, central dense	18	(2.8%)	13	(2.0%)
CC3, total central dense	22	(3.4%)	28	(4.3%)
CC4, phthisis	6	(0.9%)	3	(0.5%)
***Papillary inflammation*** c				
None (P0)	52	(8.0%)	43	(6.6%)
Mild (P1)	183	(28.2%)	200	(30.8%)
Moderate (P2)	312	(48.1%)	304	(46.8%)
Severe (P3)	102	(15.7%)	103	(15.9%)
***Conjunctival scarring*** c				
None (C0)	0	(0.0%)	1	(0.1%)
Mild (C1)	18	(2.8%)	12	(1.9%)
Moderate (C2)	400	(61.6%)	436	(67.1%)
Severe (C3)	231	(35.6%)	201	(30.9%)
***Lagophthalmos (present)***	20	(3.1%)	18	(2.8%)
**n ** ***follow-up assessments***				
0	11	(1.7%)	4	(0.6%)
1	6	(0.9%)	6	(0.9%)
2	15	(2.3%)	12	(1.8%)
3	38	(5.8%)	39	(6.0%)
4	133	(20.5%)	135	(20.8%)
5	447	(68.8%)	454	(69.9%)

a Participants with bilateral TT had one eye randomly selected as the trial eye.

b Unable to cooperate with visual acuity measurement.

c Examination of the tarsal conjunctiva was not possible in one individual.

CF, count fingers; BMI, body mass index; HM, hand movements; PL, perception of light; NPL, no perception of light; SD, standard deviation; VA, visual acuity.

Trichiatic lashes touched the cornea in 1,049 (80.7%) eyes (Table 1). Coexisting lower lid trichiasis was infrequent (13.3%). In 204 (15.7%) participants no lashes were touching the eye on the day of the baseline examination; however, all had clinical evidence of epilation of more than five trichiatic lashes. There was no significant difference in age or baseline disease severity between the 15 individuals not seen after surgery and all other participants.

### Primary Outcome

At 12-mo follow-up, recurrent trichiasis was present in 221 study eyes and a further 13 participants had undergone repeat surgery since baseline. Prevalence of recurrence was similar in the two arms of the trial (18.2% [114/628] in the polyglactin-910 group versus 19.7% [120/608] in the silk group; OR = 0.90, 95% CI 0.68–1.20). The risk difference for any recurrent trichiasis between the silk and polyglactin-910 arms was −0.016 (95% CI −0.059 to 0.023). The possibility of effect modification between arm and other factors on the primary outcome was investigated; none were significant, including surgeon.

### Secondary Outcomes

**Figure 2:**
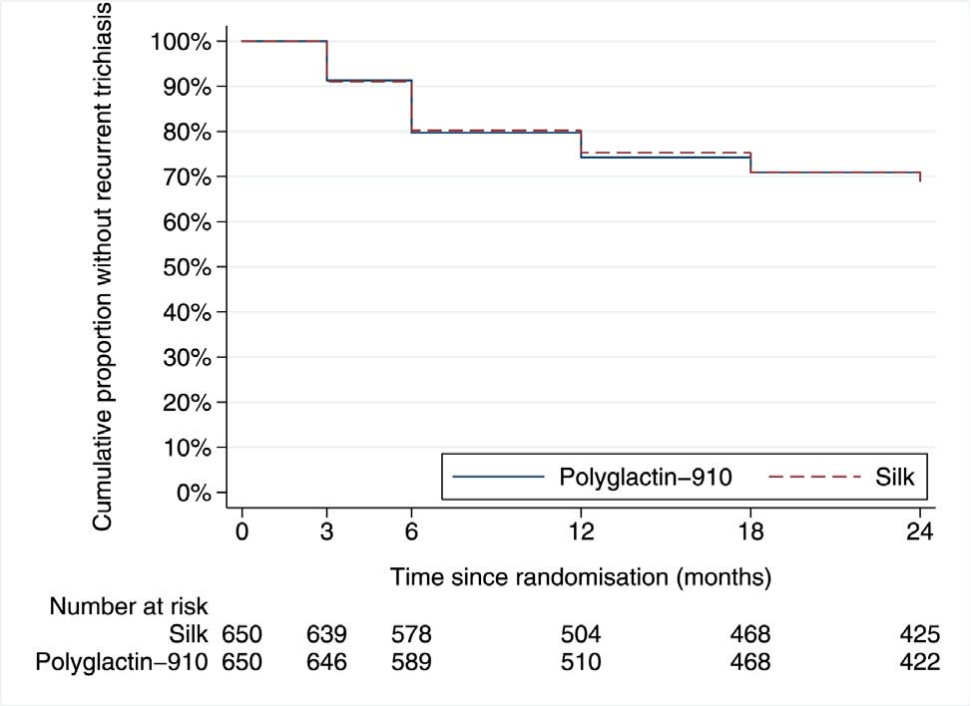
Kaplan-Meier graph of time to failure.

There was no evidence of a difference in the rate of recurrence between the two arms of the trial during the 2-y follow-up period (hazard ratio = 0.99; 95% CI 0.82–1.21) (Figure 2). Of the 236 participants seen at 6 mo but not at 3 mo, 59 (25%) had recurrent trichiasis and recurrence might have occurred earlier than 6 mo. There was no evidence of differences in the other secondary outcome measures at either 12 or 24 mo (Tables 2 and 3). Recurrent trichiasis at 1 and 2 y was associated with more severe entropion at baseline, tarsal conjunctival inflammation at baseline, increasing age, and intersurgeon variability (Table 4).

**Table 2:** Clinical characteristics of participants at 12 and 24 mo, by study arm.

Characteristic	12 mo				24 mo			
	Silk, *n* = 628		Polyglactin-910, *n* = 608		Silk, *n* = 609		Polyglactin-910, *n* = 609	
***Trichiasis (*** **n ** ***lashes)***								
No trichiasis^a^	518	(82.5%)	496	(81.6%)	491	(80.6%)	492	(80.8%)
No lashes, epilating	15	(2.4%)	22	(3.6%)	23	(3.8%)	23	(3.8%)
1–5	84	(13.4%)	77	(12.7%)	87	(14.3%)	83	(13.6%)
6–9	7	(1.1%)	7	(1.1%)	5	(0.8%)	7	(1.1%)
10–19	2	(0.3%)	2	(0.3%)	2	(0.3%)	2	(0.3%)
20+	2	(0.3%)	4	(0.6%)	1	(3.8%)	2	(0.3%)
***Best corrected LogMAR VA in trial eye***								
−0.2 to 0.3	184	(30.4%)	194	(30.9%)	167	(27.5%)	168	(27.6%)
0.3–0.7	250	(41.3%)	265	(42.3%)	257	(42.3%)	268	(44.1%)
0.7–1.1	89	(14.7%)	86	(13.7%)	107	(17.6%)	88	(14.5%)
1.1–2.0	32	(5.3%)	27	(4.3%)	27	(4.4%)	30	(4.9%)
CF/HM/PL	44	(7.3%)	48	(7.7%)	42	(6.9%)	45	(7.4%)
NPL	7	(1.2%)	7	(1.1%)	8	(1.3%)	9	(1.5%)
***LogMAR change baseline to follow-up***								
>0.3 worse	49	(8.1%)	56	(9.1%)	57	(9.4%)	60	(10.0%)
0.1–0.3 worse	97	(16.1%)	111	(17.9%)	141	(23.3%)	131	(21.8%)
Within 0.1 (same)	270	(44.9%)	270	(43.6%)	248	(41.1%)	270	(45.0%)
0.1 to 0.3 better	118	(19.6%)	114	(18.4%)	92	(15.2%)	82	(13.7%)
>0.3 better	68	(11.3%)	68	(11.0%)	66	(10.9%)	57	(9.5%)
***Entropion grade***								
0	550	(90.5%)	550	(87.6%)	538	(88.3%)	542	(89.0%)
1	34	(5.6%)	59	(9.4%)	44	(7.2%)	41	(6.7%)
2	16	(2.6%)	16	(2.6%)	22	(3.6%)	22	(3.6%)
3	6	(1.0%)	3	(0.5%)	4	(0.7%)	4	(0.7%)
4	2	(0.3%)	0	(0.0%)	1	(0.2%)	0	(0.0%)
***Corneal opacity – field grading***								
CC0, none	219	(36.0%)	246	(39.2%)	231	(37.9%)	254	(41.7%)
CC1, peripheral	138	(22.7%)	154	(24.5%)	138	(22.7%)	136	(22.3%)
CC2a, off centre faint	143	(23.5%)	121	(19.3%)	133	(21.8%)	113	(18.6%)
CC2b, off centre dense	24	(4.0%)	21	(3.3%)	27	(4.4%)	21	(3.5%)
CC2c, central faint	50	(8.2%)	52	(8.3%)	48	(7.9%)	48	(7.9%)
CC2d, central dense	21	(3.5%)	17	(2.7%)	17	(2.8%)	14	(2.3%)
CC3, total central dense	9	(1.5%)	15	(2.4%)	12	(2.0%)	21	(3.5%)
CC4, phthisis	4	(0.7%)	2	(0.3%)	3	(0.5%)	2	(0.3%)
***Corneal opacity change baseline to follow-up – photographic grading***								
Worse	11	(1.8%)	6	(1.0%)	18	(3.0%)	24	(3.9%)
No change	510	(85.0%)	531	(85.0%)	499	(82.5%)	493	(81.1%)
Better	79	(13.2%)	88	(14.1%)	88	(14.5%)	91	(15.0%)
***Corneal opacity change baseline to follow-up – field grading***								
>3 grades worse	1	(0.2%)	0	(0.0%)	1	(0.2%)	1	(0.2%)
3 grades worse	3	(0.5%)	0	(0.3%)	1	(0.2%)	1	(0.2%)
2 grades worse	10	(1.6%)	12	(1.9%)	11	(1.8%)	12	(2.0%)
1 grade worse	48	(7.9%)	53	(8.4%)	65	(10.7%)	45	(7.4%)
No change	413	(67.9%)	404	(64.3%)	375	(61.6%)	386	(63.4%)
1 grade better	93	(15.3%)	116	(18.5%)	108	(17.7%)	117	(19.2%)
2 grades better	37	(6.1%)	38	(6.1%)	43	(7.1%)	44	(7.2%)
3 grades better	2	(0.3%)	5	(0.8%)	2	(0.3%)	3	(0.5%)
>3 grades better	1	(0.2%)	0	(0.0%)	3	(0.5%)	0	(0.0%)
***Papillary inflammation***								
None (P0)	152	(25.0%)	177	(28.2%)	333	(54.7%)	343	(56.3%)
Mild (P1)	277	(45.6%)	263	(41.9%)	130	(21.4%)	115	(18.9%)
Moderate (P2)	168	(27.6%)	178	(28.3%)	136	(22.3%)	138	(22.7%)
Severe (P3)	11	(1.8%)	10	(1.6%)	10	(1.6%)	13	(2.1%)
***Lagophthalmos (present)***	5	(0.8%)	6	(1.0%)	4	(0.7%)	1	(0.2%)
***Recurrent TT by surgeon*** b								
1	31/188	(16.5%)	28/177	(15.8%)	50/181	(27.6%)	54/175	(30.1%)
2	29/127	(22.8%)	33/124	(26.6%)	50/124	(40.3%)	49/124	(39.5%)
3	17/132	(12.9%)	16/127	(12.6%)	35/130	(26.9%)	37/130	(28.5%)
4	16/138	(11.6%)	23/134	(17.1%)	36/130	(27.7%)	33/133	(24.8%)
5	21/43	(48.8%)	20/46	(43.5%)	27/44	(61.4%)	27/47	(57.5%)

a Includes individuals who had received repeat surgery, but had not developed a second episode of postoperative recurrence.

b Denominators are the number of cases performed by each surgeon, % values are the recurrences.

CF, count fingers; HM, hand movements; PL, perception of light; NPL, no perception of light; VA, visual acuity.

**Table 3:** Univariate associations between outcome measures and study arm (polyglactin-910 sutures compared to silk sutures).

Outcome	12 mo			24 mo		
	OR	95% CI	*p*-Value	OR	95% CI	*p*-Value
Recurrent trichiasis	0.90	0.68–1.20	0.48	0.99	0.78–1.25	0.90
Deterioration in visual acuity^a^	1.20	0.88–1.65	0.25	1.04	0.78–1.39	0.78
Deterioration in corneal opacity	1.02	0.70–1.47	0.93	0.73	0.51–1.05	0.09
Recurrent entropion^b^	1.34	0.94–1.93	0.11	0.94	0.66–1.33	0.72
Papillary inflammation (P2/P3)	1.02	0.80–1.31	0.85	1.05	0.80–1.36	0.74

a Deterioration of two or more lines on LogMAR visual acuity chart.

b Presence of entropion, grade 2 or more.

**Table 4:** Univariate analysis and multivariable logistic regression models for any recurrent trichiasis at 1 or 2 y following surgery.

Variable	12 mo			24 mo		
	OR	95% CI	*p*-Value	OR	95% CI	*p*-Value
**Univariate analysis**						
*Trial arm (silk)*	1.06	0.82–1.36	0.67	0.99	0.78–1.25	0.90
*Gender (female)*	0.97	0.72–1.31	0.85	1.03	0.77–1.37	0.85
*Age*	—	—	<0.0001*	—	—	<0.0001*
18–30	1	—	—	1	—	—
30–39	1.27	0.71–2.28	—	1.27	0.74–2.19	—
40–49	1.77	1.02–3.08	—	1.49	0.89–2.50	—
50–59	1.66	0.95–2.89	—	1.98	1.18–3.33	—
60–69	2.73	1.53–4.86	—	2.72	1.57–4.71	—
70+	2.17	1.02–4.59	—	2.11	1.02–4.35	—
*Entropion at baseline (grade 3/4)*	1.86	1.43–2.43	<0.0001	1.65	1.28–2.13	<0.0001
*Baseline inflammation (P2/P3)*	1.39	1.08–1.80	0.01	1.40	1.10–1.79	0.006
*Surgeon (relative to surgeon 4)*	—	—	<0.0001**	—	—	<0.0001**
1	0.99	0.68–1.44	—	1.16	0.81–1.66	—
2	1.93	1.32–2.84	—	1.87	1.28–2.72	—
3	0.87	0.57–1.32	—	1.08	0.73–1.58	—
4	1	—	—	1	—	—
5	4.86	2.92–8.09	—	4.10	2.49–6.77	—
**Multivariable logistic regression models**						
*Trial arm (silk)*	1.09	0.84–1.42	0.52	0.98	0.77–1.26	0.90
*Gender (female)*	1.08	0.79–1.49	0.62	1.18	0.86–1.60	0.30
*Age*	—	—	<0.0001*	—	—	<0.0001*
18–30	1	—	—	1	—	—
30–39	1.25	0.68–2.28	—	1.22	0.70–2.15	—
40–49	1.81	1.02–3.22	—	1.50	0.88–2.56	—
50–59	1.71	0.96–3.05	—	2.09	1.23–3.57	—
60–69	3.11	1.71–5.67	—	3.06	1.73–5.38	—
70+	2.38	1.08–5.23	—	2.30	1.08–4.89	—
*Entropion at baseline (grade 3/4)*	1.80	1.36–2.38	<0.0001	1.59	1.22–2.09	0.0006
*Baseline inflammation (P2/P3)*	1.31	1.00–1.72	0.05	1.37	1.06–1.76	0.02
*Surgeon (relative to surgeon 4)*	—	—	<0.0001**	—	—	<0.0001**
1	1.02	0.70–1.51	—	1.17	0.81–1.69	—
2	2.00	1.34–2.95	—	1.88	1.28–2.76	—
3	0.87	0.57–1.32	—	1.06	0.71–1.57	—
4	1	—	—	1	—	—
5	4.93	2.93–8.29	—	4.13	2.48–6.89	—

*p-Value for trend.

**p-Value for heterogeneity.

### Complications

Complications were observed in 219 participants (16.9%). The overall complication risk was similar in the two arms of the trial (silk 18.2% versus polyglactin-910 15.5%, *p* = 0.21; Table 5) as were risks of both intraoperative or early postoperative (7–10 d) complications (Table 5). There was some evidence that some late complications (after 7–10-d follow-up) were seen less frequently in the polyglactin-910 group than the silk group: conjunctival granulomas (OR = 0.63, 95% CI 0.40–0.99, *p* = 0.045) and suture fragments in situ (OR = 0.06, 95% CI 0.01–0.48, *p* = 0.008).

**Table 5:** Intra- and postoperative complications, by study arm.

Complication	Silk		Polyglactin-910		*p*-Value
	*n*	Percent	*n*	Percent	
***Any complication***	118	(18.2)	101	(15.5)	0.21
***Intraoperative***					
Bleeding	6	(0.9)	12	(1.9)	0.24^a^
***Early (by 7–10 d)***					
Infection/erythematous swelling/conjunctivitis	7	(1.1)	3	(0.5)	0.34^a^
***Late (after 7–10-d follow-up)***					
Granuloma	51	(8.7)	34	(5.7)	0.04
Notching	70	(11.9)	84	(14.0)	0.29
Residual suture fragments	15	(2.6)	1^b^	(0.2)	<0.001^a^
Infection	0	(0.0)	3	(0.6)	0.12^a^

b Undissolved suture identified at 3 mo.

a Fisher exact test.

## Discussion

In this randomised controlled trial we tested the hypothesis that absorbable sutures (polyglactin-910) reduce the recurrence of trichiasis a year after surgery, compared with standard silk sutures removed at 7 to 10 d. We found no evidence of a difference in recurrence risk at either the primary endpoint (1 y) or over the 2-y follow-up period. The risk difference between arms in the primary end point was very small (1.1%) with narrow confidence intervals (−3.9% to 6.0%). This finding is consistent with the two sutures being equivalent from a clinical perspective, although the trial was not designed to test this hypothesis.

The relationship between suture type and trichiasis recurrence was examined in a nonrandomised study from Egypt, which found a significantly lower recurrence rate in cases where absorbable sutures were used [11]. However, this lower rate may have been partly due to confounding, as individuals who received absorbable sutures were treated in the private sector, and environmental and socioeconomic factors might have led to more severe preoperative disease and increased risk of TT recurrence amongst those treated in the public sector.

Our results suggest that, despite the lack of difference in recurrence risk, there are operational reasons why a trachoma control program might consider using absorbable sutures such as polyglactin-910. Firstly, most individuals requiring TT surgery live in relatively remote rural settings, and many are relatively old and with limited financial resources. Under such conditions transport may be limited or a significant financial burden, presenting a major challenge for the patient to reattend a distant health facility for suture removal. The use of absorbable sutures would eliminate the need for an early postoperative review at 7 to 10 d, allowing the service to focus on a more important 3- to 6-mo review to check for recurrent trichiasis and provide repeat surgery. Secondly, because not all patients will attend for routine follow-up after surgery, retained sutures are not uncommon and can cause significant trauma to the cornea. Even with the considerable logistical resources (vehicles and personnel) available in this trial and the high level of engagement by the participants in the follow-up, we still found that 2.6% of people in the silk arm had unremoved sutures at 3 mo. It is likely that this problem is significantly more frequent under operational conditions. The use of absorbable sutures generally avoids this problem. Whether a trachoma control program adopts the use of absorbable sutures for trichiasis surgery may be influenced by several locally specific considerations, including the relative local costs of different sutures types and the potential cost and time savings of not having to arrange for early review and suture removal.

The trichiasis recurrence risk in our trial is at the lower end of the range typically reported; 19% (234/1,236) of the trial eyes reexamined at 1 y had recurrent TT. Most trials or consecutive series have reported similar 1-y TT recurrence risks in the range of about 20% to 40% at 1 y [5]–[8],[11],[12],[15]. An exception to this was the STAR trial, also conducted in Ethiopia, which reported a notably lower cumulative 1-y recurrence risk of 6.9% to 10.3%, depending on intervention arm [13].

The reasons for recurrence are multiple. The time profile of recurrence suggests that there are two distinct phases: (1) early recurrence, occurring during the first 6 mo; (2) late recurrence, which occurs from 6 mo onwards and develops at a reduced rate. In the few prospective trials or case series with a year or more follow-up, early recurrence has tended to account for the majority of cases [5],[8],[9],[12],[13]. It is generally thought that this early recurrence is largely determined by the preoperative disease severity and how the surgery is performed in terms of both type and quality of procedure.

We found that recurrent trichiasis at both 1 and 2 y was significantly associated with both more severe entropion at baseline and baseline conjunctival inflammation. Several studies have found a consistent relationship between the severity of preoperative disease (usually in terms of numbers of lashes or entropion severity) and the risk of recurrence [8],[12],[13]. More severely scarred eyelids are technically more challenging to operate on and therefore the correction of the anatomical abnormality may be less satisfactory or there may be a more active underlying profibrotic disease state.

The type of operation performed also has a major effect on the outcome. A systematic review of the alternative procedures that have been compared in clinical trials found evidence to support the use of procedures that involve a full thickness incision through the tarsal plate, rotation of the distal fragment, and fixation with everting sutures [4]. There are two commonly performed alternative procedures: (1) BLRT and (2) PLTR. These procedures have been compared head to head in a single trial with only relatively short follow-up, which found equivalent recurrence rates [23]. The WHO recommends the use of the BLTR in its literature, but acknowledges that some programmes have chosen to train their surgeons to use alternative procedures (such as the PLTR), and that a switch to the BLTR is therefore not advised [3]. A large-scale randomised trial with long-term follow-up of these two major alternatives in clinical practice would be of value.

The quality of the surgery is a major determinant of outcome. We found significant variation in the recurrence risk between surgeons. Surgeons were assigned to operate on patients on a “first-come-first-served” basis; therefore, it is unlikely that there was selection bias in the severity of cases operated on by specific surgeons in this trial. Moreover, intersurgeon variation persisted after adjusting for baseline disease severity in the model. Several other studies have found significant variation between surgeons, suggesting that there might be subtle variations in technique that influence the risk of recurrence [8],[10],[23]. Short incisions probably lead to inadequate eversion and early recurrence; it has been suggested that an incision of at least 20 mm should be used [24]. The degree of eversion at the end of the operation, regulated by the positioning and tension of sutures, is probably a key determinant of recurrent TT.

Late recurrent TT might occur owing to the immuno-fibrogenic factors that caused conjunctival scarring and TT in the first place, rather than problems directly related to the surgical procedure [25]. A number of trials in different settings have investigated whether the use of postoperative azithromycin can affect the outcome. A study from Ethiopia found a significant reduction in recurrence after antibiotic use whilst a study from The Gambia did not. This difference might have been in part due to the difference in exposure to *Chlamydia trachomatis* infection between participants in the different trials [8],[13].

Complications of trichiasis surgery are uncommon. There were a few cases with moderate bleeding during surgery, all of whom were managed successfully with firm pressure on the eyelid for a few minutes. Similarly, postoperative wound infections were uncommon and resolved rapidly with antibiotics. The only notable differences between the two trial arms occurred later: retained suture and conjunctival granulomas (large enough to obscure vision) were more common in lids where silk sutures had been used. Silk is recognised to be quite proinflammatory in operative wounds, which might explain the higher granulomas rate.

This study has a number of strengths including the large size, high follow-up rates, good balance of clinical and demographic characteristics between the randomisation groups, the fact that observations at each time point were made by a single observer, and that outcome measures were determined by masked individuals. There were slightly more individuals in the polyglactin-910 group who were lost to follow-up at 12 mo; however, most of these participants had moved out of the study area and so this small difference is not attributable to a treatment effect. Despite careful standardisation procedures, there was still some variation between the results of the different surgeons, indicating the importance of how the surgery is performed. These may include subtle variation in the length of the incision or the tension on the everting sutures. Individuals with minor trichiasis were excluded from this trial and enrolled into a separate trial evaluating the efficacy of surgery versus epilation. However, we are unaware of any reason to think that these findings could not be generalised to the full range of trichiasis severity. It would be interesting to know if there were significant differences in the participants' experience of the different sutures used in this trial and their perspectives on the potential benefit of not needing to have the absorbable sutures removed; however, we did not formally investigate this. Detecting differences in changes in both corneal disease and visual acuity would probably require a longer follow-up period.

Polyglactin-910 absorbable sutures had a similar risk of trichiasis recurrence to silk sutures for PLTR TT surgery and comparable secondary outcomes. However, from a programmatic perspective, polyglactin-910 offers the major advantage that patients do not have to be seen soon after surgery for suture removal. The postoperative review can be delayed for 3–6 mo, which might allow us to better determine who needs additional surgery and offset the slightly higher cost of absorbable sutures. The logistical advantages of using absorbable sutures should be taken into consideration when considering the choice of suture material.

## Supporting Information

Table S1 **(a) Expanded grading system for entropion, conjunctivilisation and CO. (b) Conjunctivilisation: anterioplacement of the muco-cutaneous junction of the upper eyelid. (c) Corneal scarring (assessed with eye in primary position).**
(DOCX)

Text S1 **Trial protocol.**
(DOC)

Text S2 **CONSORT checklist.**
(DOC)

## Abbreviations

BLTR: bilamellar tarsal rotation
CC: degree of central corneal scarring
CO: corneal opacification
OR: odds ratio
PLTR: posterior tarsal rotation
TT: trachomatous trichiasis
WHO: World Health Organization
