# Supplementary material for: Absorbable Versus Silk Sutures for Surgical Treatment of Trachomatous Trichiasis in Ethiopia: A Randomised Controlled Trial
Source: PLoS Med. 2011 Dec 13;8(12):e1001137. doi: 10.1371/journal.pmed.1001137 (PMC3236737; doi:10.1371/journal.pmed.1001137)
Supplement: Table S1 — (a) Expanded grading system for entropion, conjunctivilisation and CO. (b) Conjunctivilisation: anterioplacement of the muco-cutaneous junction of the upper eyelid. (c) Corneal scarring (assessed with eye in primary position). (DOCX) [file pmed.1001137.s001.docx]

**(a) Expanded grading system for entropion, conjunctivilisation and corneal opacification.**

| **Sign** |  | **Definition** |  |  | |
| --- | --- | --- | --- | --- | --- |
| ***Entropion (assessed with eye in primary position)*** | | |  | |  |
| E0 | (none) | None |  | | No WHO grading system for entropion |
| E1 | (mild) | <50 % of lid margin rolled inwards without cornea - lash base contact | | |  |
| E2 | (moderate) | >50% of lid margin rolled inwards without cornea –lash base contact | | |  |
| E3 | (severe) | Lid margin rolled inwards with <50% lash base – cornea contact | | |  |
| E4 | (severe) | Lid margin rolled inwards with >50% lash base – cornea contact | | |  |

**(b) Conjunctivilisation: anterioplacement of the muco-cutaneous junction of the upper eyelid**

| **Sign** | **Definition** |  |
| --- | --- | --- |
| 0 | None. The muco-cutaneous junction is posterior to the line of Meibomian gland orifices, which clearly lie within the lid margin skin. | 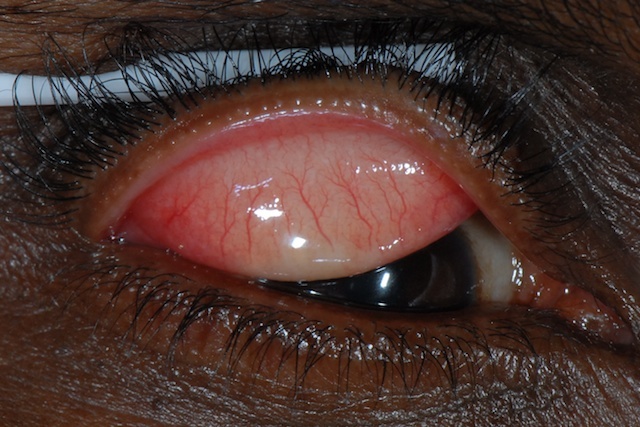 |
| 1 | Muco-cutaneous junction is located anterior to its normal position; the junction is very close to Meibomian gland orifices, which lie just within lid margin skin. | 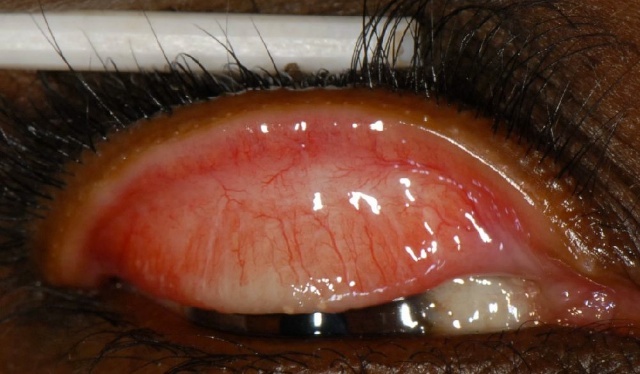 |
| 2 | The muco-cutaneous junction is located anterior to the line of the Meibomian gland orifices for <50% lid (in this picture temporal orifices are enveloped in conjunctival epithelium) | 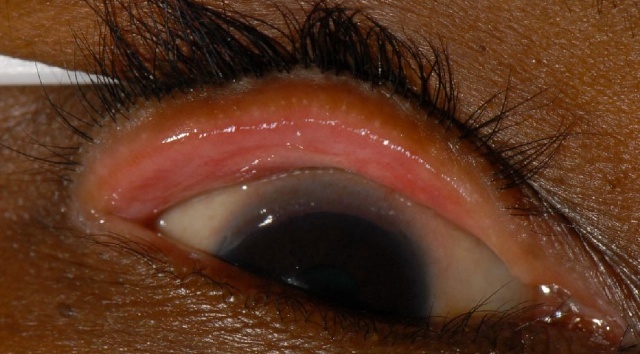 |
| 3 | The muco-cutaneous junction is located anterior to the line of the Meibomian gland orifices for >50% lid (in this picture all central orifices are enveloped in conjunctival epithelium) | 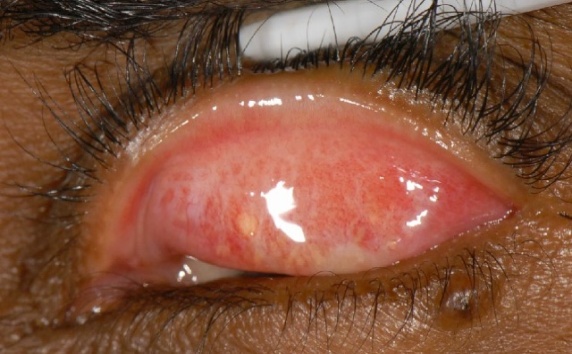 |

**(c) *Corneal scarring (assessed with eye in primary position)***

|  | | | **WHO equivalent grade** | |
| --- | --- | --- | --- | --- |
| CO-0 |  | None |  | CC0 |
| CO-1 | Location: | Opacity, outside central 4mm of cornea |  | CC1 |
|  | Density: | Any |  |  |
| CO-2a | Location: | Within central 4mm, but not entering central 1mm. |  | CC2 |
|  | Density: | Pupil margin visible through opacity. |  |  |
| CO-2b | Location: | Within central 4mm, but not entering central 1mm. |  |  |
|  | Density: | Pupil margin not visible through opacity. |  |  |
| CO-2c | Location: | Overlying central 1mm, i.e. visual axis |  |  |
|  | Density: | Pupil margin visible through opacity. |  |  |
| CO-2d | Location: | Overlying central 1mm, i.e. visual axis |  |  |
|  | Density: | Pupil margin not visible through opacity. |  |  |
| C0-3 | Location: | All of central 4mm |  | CC3 |
|  | Density: | Pupil margin not visible through opacity |  |  |
| CO-4 |  | Phthisis |  |  |
